# The ReIMAGINE Multimodal Warehouse: Using Artificial Intelligence for Accurate Risk Stratification of Prostate Cancer

**DOI:** 10.3389/frai.2021.769582

**Published:** 2021-11-16

**Authors:** Aida Santaolalla, Tim Hulsen, Jenson Davis, Hashim U. Ahmed, Caroline M. Moore, Shonit Punwani, Gert Attard, Neil McCartan, Mark Emberton, Anthony Coolen, Mieke Van Hemelrijck

**Affiliations:** ^1^ King’s College London, School of Cancer and Pharmaceutical Sciences, Translational Oncology and Urology Research (TOUR), London, United Kingdom; ^2^ Philips Research, Department of Hospital Services and Informatics, Eindhoven, Netherlands; ^3^ Philips, Data Science Services, Best, Netherlands; ^4^ Imperial College London, Faculty of Medicine, Imperial Prostate, Department of Surgery and Cancer, London, United Kingdom; ^5^ Division of Surgical and Interventional Science, University College London, London, United Kingdom; ^6^ Centre for Medical Imaging, University College London, London, United Kingdom; ^7^ Cancer Institute, University College London, London, United Kingdom; ^8^ Department of Biophysics, Donders Institute, Radboud University Nijmegen, Nijmegen, Netherlands

**Keywords:** prostate cancer, risk stratification, artificial intelligence, data warehouse, database, data management, data integration, data science

## Abstract

**Introduction.** Prostate cancer (PCa) is the most frequent cancer diagnosis in men worldwide. Our ability to identify those men whose cancer will decrease their lifespan and/or quality of life remains poor. The ReIMAGINE Consortium has been established to improve PCa diagnosis.

**Materials and methods.** MRI will likely become the future cornerstone of the risk-stratification process for men at risk of early prostate cancer. We will, for the first time, be able to combine the underlying molecular changes in PCa with the state-of-the-art imaging. ReIMAGINE Screening invites men for MRI and PSA evaluation. ReIMAGINE Risk includes men at risk of prostate cancer based on MRI, and includes biomarker testing.

**Results.** Baseline clinical information, genomics, blood, urine, fresh prostate tissue samples, digital pathology and radiomics data will be analysed. Data will be de-identified, stored with correlated mpMRI disease endotypes and linked with long term follow-up outcomes in an instance of the Philips Clinical Data Lake, consisting of cloud-based software. The ReIMAGINE platform includes application programming interfaces and a user interface that allows users to browse data, select cohorts, manage users and access rights, query data, and more. Connection to analytics tools such as Python allows statistical and stratification method pipelines to run profiling regression analyses.

**Discussion.** The ReIMAGINE Multimodal Warehouse comprises a unique data source for PCa research, to improve risk stratification for PCa and inform clinical practice. The de-identified dataset characterized by clinical, imaging, genomics and digital pathology PCa patient phenotypes will be a valuable resource for the scientific and medical community.

## Introduction

Prostate cancer is the most frequent cancer diagnosis in men worldwide and the second most frequent cause of death in men worldwide after lung cancer (Bray et al., 2018; Sung et al., 2021). One in eight men will be diagnosed with prostate cancer during their lifetime. Despite the high incidence rate, the 5-years survival rate for prostate cancer is over 90%, given that most of the cancers diagnosed are confined within the prostate (locally or regionally) and only about 7% of men will have more advanced prostate cancer at the time of diagnosis (Rawla, 2019). Despite this, our ability to identify those men whose cancer will decrease their lifespan and/or quality of life remains poor (Bangma et al., 2007). Currently, the established diagnostic pathway for prostate cancer consists of PSA screening followed by diagnostic biopsy which presents high rates of over-diagnosis (1.7–67%), over-treatment, missed diagnoses, and poor risk-stratification (Loeb et al., 2014). These errors result in the application of treatments that have little or no benefit, the reduction of the quality of life of patients, and the inefficient use of valuable healthcare resources (Klotz, 2013). Prostate Cancer risk assessment tools to discriminate those men at lowest risk from those at highest risk of aggressive disease at diagnosis are necessary to improve patient outcomes and quality of life.

The ReIMAGINE Consortium (ReIMAGINE Consortium, 2021), which consists of three academic partners (University College London (UCL), King’s College London (KCL), and Imperial College London), several commercial partners and a Patient and Public Involvement (PPI) Sub-Committee, has been established to undertake discovery that will correct the ongoing key errors in the PCa diagnostic pathway (over-diagnosis, over-treatment, missed-diagnoses, and poor risk-stratification). For the first time, we will combine the underlying molecular changes in the cancer with deep clinical phenotyping and the state-of-the-art imaging. In the future, this may allow us to predict prostate cancer status for the individual (low, medium or high risk) without recourse to biopsy, and to foresee which prostate cancers will be more likely to progress over time, something that has largely eluded us. Therefore, men will be subject to fewer but better biopsies; effective risk stratification strategies will lead to appropriate treatment allocation, ultimately improving quality of care and increasing the cost-effectiveness of healthcare systems.

Projects such as ReIMAGINE which need to integrate large datasets require an integrated data warehouse. This includes capacity for not only a large number of patients (“long data”), but also with a large number of data fields from different data types (“wide data”) (Hulsen and Moustafa, 2021). The data warehouse needs to be able to store the raw data in combination with metadata that can be queried to enable e.g., cohort selection, data visualization and statistical analysis. The data also needs to be FAIR (Wilkinson et al., 2016)—findable, accessible, interoperable and reusable—to comply with the latest regulations. In the open-source world, there are several systems that can act as a data warehouse for clinical data. One of these is tranSMART, a web-based platform for the integration of data created for translational research (Scheufele et al., 2014). TranSMART supports several statistical analyses, such as correlation analysis, logistic regression, and survival analysis (Kaplan-Meier plots). Genomics data can be analyzed in tranSMART as well, through built-in analysis methods such as group tests and heatmaps. Another open-source data platform is i2b2 (Informatics for Integrating Biology and the Bedside) (Murphy et al., 2010). i2b2 is used at over 250 locations worldwide. i2b2 enables sharing, integration, standardization, and analysis of heterogenous data from healthcare and research. Another web-based data platform is cBioPortal (Gao et al., 2013), which was created to store, visualize and analyze large-scale cancer genomics datasets in combination with clinical data. Outside of the open-source world, there are also some solutions available created by companies active in healthcare IT such as Amazon [Amazon HealthLake (Amazon (2021)] Google [Google Cloud Healthcare Data engine (Google (2021)] and Microsoft [Microsoft Cloud for Healthcare (Microsoft (2021)]. In comparison to the open-source tools, these proprietary data warehouses often have the advantage of ISO certifications and stricter compliance to privacy regulations. These data warehouses need to support healthcare data standards such as DICOM and HL7/FHIR, and connect seamlessly to statistical analysis tools and programming languages such as SPSS, R and Python for downstream analysis. For these “big data” projects, the date warehouse needs to provide data to AI frameworks such as Tensorflow, Keras and PyTorch, to be able to fully exploit the wide range of possibilities that AI and statistical machine learning offer: automated prostate segmentation (Ghavami et al., 2019), risk stratification (Varghese et al., 2019), diagnosis (Yoo et al., 2019), feature selection (Sechidis et al., 2019), decontamination for informative covariate missingness or informative censoring (Wulaningsih et al., 2015; Häggström et al., 2018), treatment responder identification (Ubels et al., 2020), and more. However, in most data warehouses this connection is either missing or not very well executed. The data warehouse that we will use for the ReIMAGINE project is ISO certified, complies with all privacy regulations such as GDPR, has built-in support for DICOM and HL7/FHIR, and connects seamlessly with Python (including Tensorflow, Keras and PyTorch) through the PyCDaL module as well as the statistical software SaddlePoint-Signature and SaddlePoint-Mosaics.

The manuscript is structured in the following order: in the materials and methods section, we describe how the ReIMAGINE project is setup, how we have created the ReIMAGINE Multimodal Warehouse and how AI tools are being developed on top of this warehouse. In the results section, we describe what data is being collected and how it is made available in the warehouse. In the discussion section, we put our research in a wider context.

## Materials and Methods

### ReIMAGINE Project

The multidisciplinary ReIMAGINE consortium was constituted to improve prostate cancer stratification and risk prediction at diagnosis. The vision of the ReIMAGINE project is built on our previous work (Ahmed et al., 2017) which has shown that magnetic resonance imaging (MRI) of the prostate was twice as good at identifying men at risk as the standard practice, and moreover rarely missed men with potentially lethal disease. Moreover, the implementation of high-quality multiparametric magnetic resonance imaging (mpMRI) in the baseline diagnostic pathway can improve the detection rate of clinically significant cancer and can effectively prevent biopsy in a proportion of men at low risk of PCa (Ahmed et al., 2017; Fulgham et al., 2019; National Institute for Health and Care Excellence, 2019). Therefore, mpMRI will likely become the future cornerstone of the risk-stratification process for men at risk of early prostate cancer (Eklund et al., 2021). However, current stratification tools do not benefit from the state-of-the-art imaging and a multimodal approach combining mpMRI with a deeply characterized patient phenotype is still not fully investigated.

ReIMAGINE developed two work strands (WS) to explore multimodal risk stratification strategies: the ReIMAGINE Prostate Cancer Screening Study and the ReIMAGINE Prostate Cancer Risk Study, which together will collect and generate clinical, imaging, pathology, and omics data from 1,300 individuals. The ReIMAGINE Prostate Cancer Risk Study (NCT04060589) (Marsden et al., 2021a), establishes the first cohort of 1,000 men who undergo diagnostic tests for suspected prostate cancer by means of an mpMRI-based pathway. These men donate baseline healthcare data, blood, urine, prostate tissue, imaging and digital pathology for marker analysis, after appropriate consent. They are being recruited in three major centres in London with high quality mpMRI systems available. The ReIMAGINE Prostate Cancer Screening Study (NCT04063566) (Marsden et al., 2021b), PubMed ID 34593491 established a screening cohort which tests the performance of bpMRI in a randomly invited general practitioner (GP) population: 300 men, between the ages of 50–75 years, with no previous PCa diagnosis. This allows us to predict the prevalence of men at risk within the community. The projects generate large volumes of multimodal data (clinical information, imaging data, digital pathology data (the risk study only), as well as information from markers in blood, tissue, and urine (the risk study only)) that will be linked for each patient and integrated in a searchable warehouse facilitating the analytical phase using advanced mathematical techniques.

### ReIMAGINE Warehouse and Clinical Data Lake

The ReIMAGINE large multimodal data repository requires a robust, secure, and effective warehouse to facilitate scientific discovery and enable the use of AI technologies to develop accurate risk stratification tools for prostate cancer.

**FIGURE 1:**
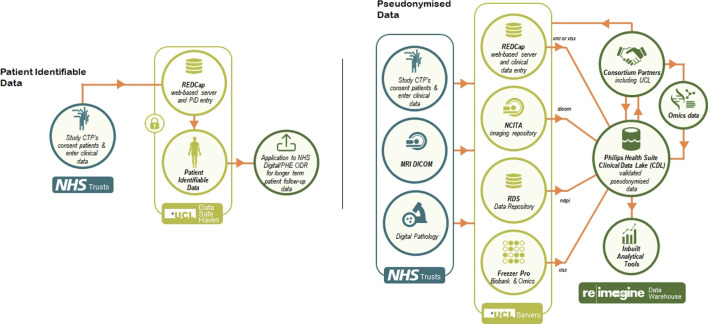
The ReIMAGINE data management infrastructure.

To allow for the development of the warehouse, Philips joined ReIMAGINE given their previous experience with large consortium datasets such as the Movember Foundation’s GAP3 cohort (Hulsen et al., 2016). Philips has specifically implemented a platform for ReIMAGINE that provides a secure environment to host deidentified patient information and enables secure standard protocols for bulk transfer of data, integrates, and harmonizes all data types via Study ID. Moreover, the platform allows for cohort selection, provides project descriptive statistics and allows for creation of sub studies to allow for restricted access to each of the partners in the consortium (Figure 1).

More specifically, to store all data from both work strands of ReIMAGINE, we created an instance of the Clinical Data Lake (CDL) (Hulsen et al., 2021) of the Philips HealthSuite Digital Platform (HSDP) (Philips (2021)), consisting of cloud-based software that can be run through any major web browser. This CDL has been designed to conform to all current healthcare security and privacy requirements. Data curation packages allow full validation, harmonisation and annotation of collected data. The platform has built-in support for the DICOM and FHIR standards, custom data types (e.g. clinical, omics and biosamples) as well as hierarchical access and logging of data processing activities. The ReIMAGINE platform includes application programming interfaces (APIs) and a user interface (UI). The APIs will be used to connect with other systems and analysis scripts. The UI allows users to browse data, select cohorts, view MR and digital pathology images, manage users and access rights, query data, and more. Connection to analytics tools such as Python and R (including AI frameworks such as Tensorflow) allows statistical and stratification method pipelines to run profiling regression analyses. The platform supports the deployment of Interpretable Artificial Intelligence (IAI) and Bayesian inference methods for rapid and scalable risk stratification of prostate cancer. These algorithms will include novel findings around overfitting of data (Coolen et al., 2017; Coolen et al., 2020) and latent class models (Rowley et al., 2017) which will help us to stratify patients more correctly.

### AI Tools Developed for ReIMAGINE

Clearly, one would want to use and integrate all patient measurements (from deep phenotyping and imaging) simultaneously for predicting an individual’s prostate cancer status. However, in projects such as ReIMAGINE, predictive analysis and regression protocols and pipelines require nonstandard approaches to handle the mismatch between the large number and richness of available measurements relative to the size of the trial. In such scenarios, conventional statistical and AI methods are in danger of overfitting. This manifests itself in excellent performance of one’s data analysis pipeline in predicting cancer progression risk on the processed data, which fails to be reproduced when applied to previously unseen patients. In addition, our methods need to handle and integrate effectively the distinct characteristics of the different data modalities (imaging markers, blood biomarkers, clinical variables, genomic variables, etc), as well as the potential latent heterogeneity of the patients and their disease. To address these combined challenges of high data dimensionality, covariate disparity, and latent cohort heterogeneity, we build a data analytics pipeline (based on the libraries underlying the SaddlePoint-Signature and SaddlePoint-Mosaics software packages https://www.saddlepointscience.com/) which combine cross-validation protocols, optimisation tools for covariate selection, and modern mathematical techniques with which to “decontaminate” regression outcomes for the effects of overfitting [see e.g. (Coolen et al., 2017; Sheikh and Coolen, 2019; Coolen et al., 2020)], with the use of modality-specific “meta-covariates”. The latter are personalised and optimised modality-specific risk scores (decontaminated for overfitting), which are subsequently used as integrated digital biomarkers that capture the relevant predictive information in each of the data sources. The dimension reduction thus achieved, without sacrificing predictive information (as would have been the case with principal component analysis type methods), enables clinically relevant latent heterogeneity (if present) to be mapped and used for further individualisation of prostate cancer status prediction. The AI/statistical analysis methods used will be Bayesian in nature, and hence include reliability estimates of predictions, and be fully interpretable.

## Results

The ReIMAGINE multimodal dataset has so far acquired, the complete cohort for ReIMAGINE Prostate Cancer Screening Study (NCT04063566) (Marsden et al., 2021b), which includes detailed clinical phenotype (over 250 parameters per participant containing demographics, clinical baseline characteristics, bpMRI information, secondary care MRI and biopsy information) and bpMRI dicom images for 300 men randomly invited through general practitioner (GP). The ReIMAGINE Screening data has undergone quality assurance and quality control (QA&QC) before data soft lock with excellent results consistent across different data parameters (overall percentage of error rate of 0.52). The analyses of ReIMAGINE Screening data are ongoing, with the aim of communicating the primary outcomes in Q4 2021. Currently, the ReIMAGINE Prostate Cancer Risk Study (NCT04060589) (Marsden et al., 2021a), has recruited 773 men and aims to reach 1,000 men in Q1 2022. ReIMAGINE Risk has collected detailed clinical phenotype for each recruited man (over 350 parameters per participant containing demographics, clinical baseline characteristics, mpMRI information, biopsy information, TNM and biological material information), alongside annotated mpMRI DICOM images and high-resolution digital pathology. To QA&QC the high-resolution digital pathology, a random selection of 30 patients’ digital pathology images, from multiple sites, were sent to a ReIMAGINE commercial partner to assess the quality of the images and robustness of the scanning SOP. The slides were verified to be of the correct scanning profile and appropriate quality. With a withdrawal rate of less than 1% this means the total figure for each project remains very close to the recruitment figure itself. Moreover, a biosample repository of urine, blood, tissue and generated omics, has been created, which after processing and aliquoting has resulted in over 40,000 biospecimens available. All the data will be uploaded and integrated in the platform in Q4 2022.

**FIGURE 2:**
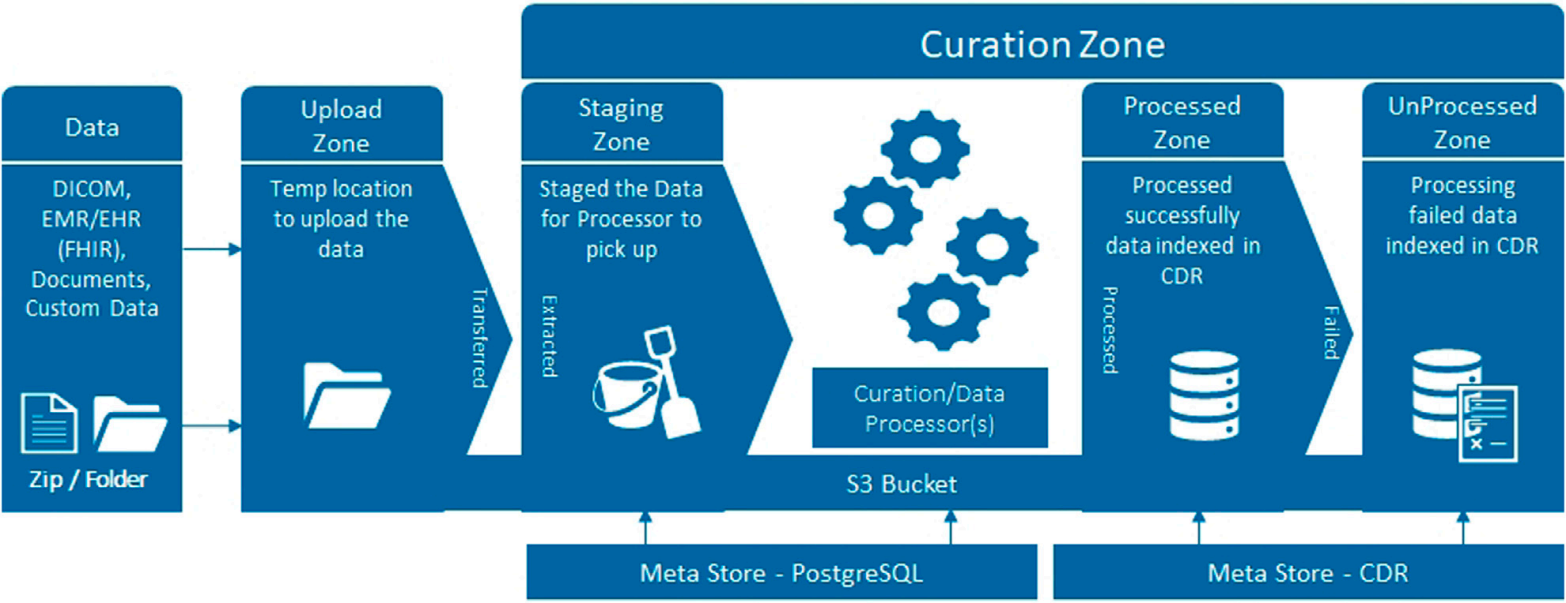
Schematic diagram of the upload workflow of the CDL.

Several terabytes of baseline clinical information, genomics, blood, urine, fresh prostate tissue samples, digital pathology and radiomics data will be analysed. Data output, as well as a large part of the raw data, will be de-identified, stored with correlated mpMRI disease endotypes and, in the future, linked with long term follow-up outcomes in an instance of the Philips HSDP CDL. Data curation packages allow full validation, harmonisation and annotation of collected data. The platform has built-in support for the DICOM and FHIR standards (Figure 2), as well as hierarchical access and logging of data processing activities. The ReIMAGINE APIs will be used to connect with other systems and analysis scripts. The UI allows users to browse data, select cohorts, view MR and digital pathology images, manage users and access rights, query data, and more. Connection to analytics tools such as Python and R allows statistical and stratification method pipelines to run profiling regression analyses.

**FIGURE 3:**
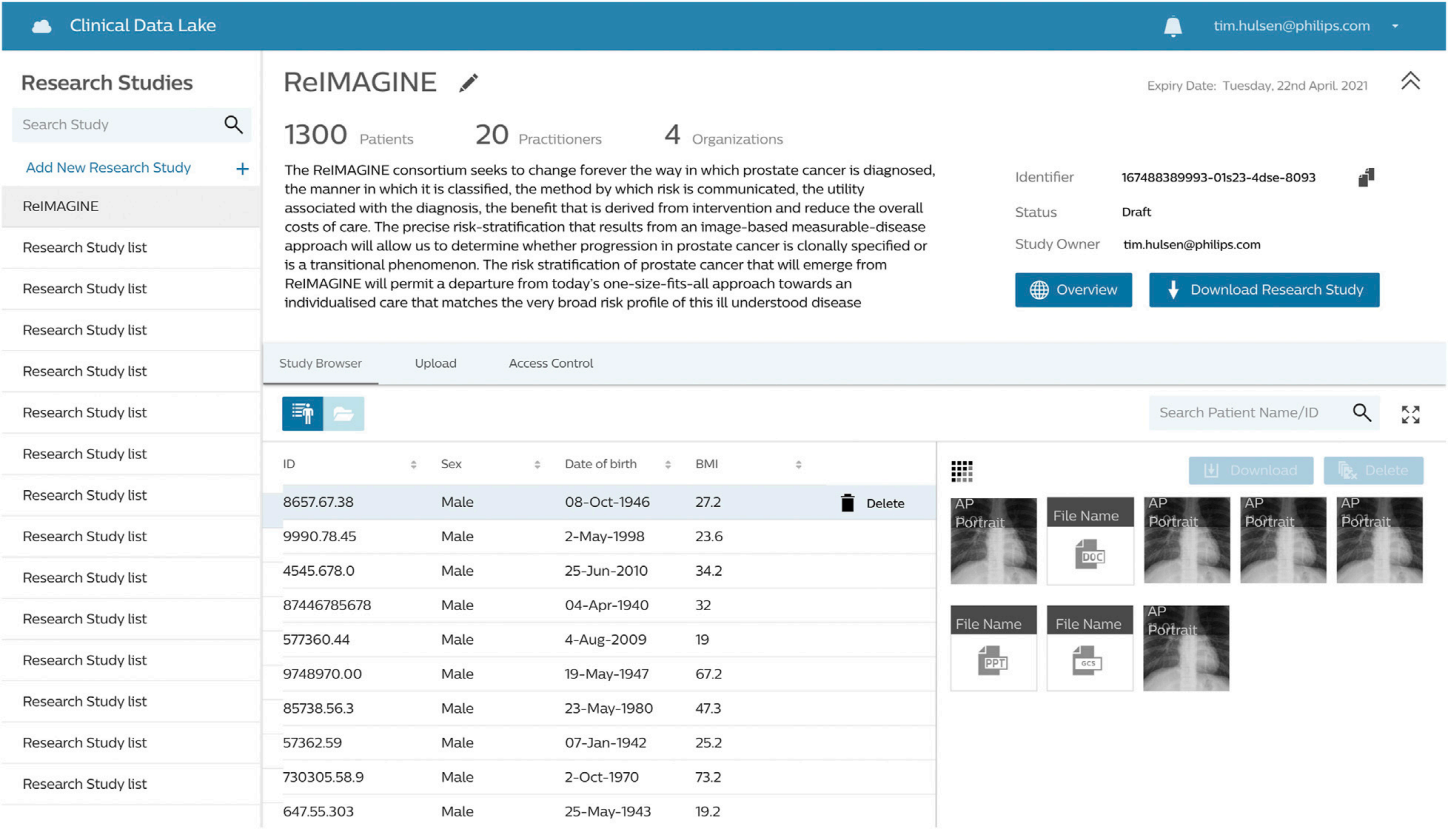
The ReIMAGINE database in the CDL user interface. All data displayed is synthetic data.

The ReIMAGINE database in the Clinical Data Lake (Figure 3) is organized using several levels: work strand, data type and subject. Data from each workstrand is uploaded separately with the metadata “WS1” or “WS2” to keep the data separated. Data is organized according to the functionality of the Clinical Data Lake: DICOM (as well as FHIR) data can be processed automatically and is thus uploaded separately from clinical data and genomics data, which need custom data types. Each data item is connected to a subject ID: in DICOM files, this is DICOM tag 0010.0020; in other files there is a field names ‘subject ID’ which contains the subject identifier. After ingestion into the Clinical Data Lake, data can be queried using SQL queries or FHIR queries. The Clinical Data Lake user interface will also have the functionality to browse through patient cohorts.

The data in the ReIMAGINE warehouse is available to all consortium partners. After completion of the project, the data will also be available to the broader clinical and scientific community via request to the ReIMAGINE Biological Research Committee (BRC). The BRC has the responsibility to develop and define rules for access to collaboration materials, clinical samples generated and collected and data complying with the Funder guidelines.

## Discussion

The ReIMAGINE deeply phenotyped cohort, comprising MRI-derived prostate cancer endotypes, will enable the correction of 40 years of risk-stratification error in early prostate cancer. The ReIMAGINE Multimodal Warehouse will be a rich data source for prostate cancer research, well-suited to improve stratification strategies for prostate cancer and inform clinical practice to ultimately improve patient care, similar to other large prostate cancer projects such as the Movember GAP3 project (Bruinsma et al., 2018). It is constructed in order to be able to answer the specific research questions of the ReIMAGINE project in a clear and reproducible manner. However, because of its availability in a data warehouse on the internet and its adherence to the FAIR guidelines (Wilkinson et al., 2016), it can be reused for other purposes as well. The ReIMAGINE consent forms include an opt-in for the reuse of data in future research projects. In contrast to many of the open-source data warehouses, the infrastructure underlying the CDL is ISO certified for compliance to security and privacy regulations Philips (2021).

Based on the nature of the study, the data collection has been tailored to improve the restratification in early detection of prostate cancer. For that reason, the usability of the dataset for other purposes could be limited. Furthermore, the study population has been consented in several primary and secondary NHS centers in London. The study has strong internal validity, and the study population is representative for the UK population. However, it might not be generalizable to other countries.

In the future, after completion of the study, the data in the warehouse will be accessible for the scientific community to answer other questions in the field of prostate cancer, such as how to exploit the possible prognostic or predictive value of longitudinal data acquired during follow-up, how personalised predictive models could be used in ‘reverse mode’ to recommend individualised treatment pathways, or what novel clinical insights could be extracted from causal inference techniques. Besides its application in the area of prostate cancer, the construction of the warehouse also gives insights on how to build a ‘big data’ warehouse in such a way that it can be easily connected to the latest AI algorithms. It can become the standard for scientific projects that combine multimodal data (clinical, imaging, genomics, digital pathology, etc.) to come to new insights using AI.

## The ReIMAGINE Study Group

Eric Aboagye, Hashim U. Ahmed, Fatima Fatima, Bana Ambasager, Gerhardt Attard, Teresita Beeston, Mariana Bertoncelli, Charlotte Bevan, Heather Bholastewart, Paul Boutros, Giorgio Brembilla, Louise Brown, Ton Coolen, Anthony Coolen, Ged Corbett, Jenson Davis, Caroline Dive, Eytan Domany, Mark Emberton, Elena Frangou, Andrew Feber, Francesco Giganti, Miriam Goncalves, Fiona Gong, Saran Green, Joanna Hadley, Ashling Henderson, Ralf Hoffmann, Tim Hulsen, Elizabeth Isaac, Richard Kaplan, Sarp Keskin, Douglas Kopcke, Natasha Majid, Teresa Marsden, Malcolm Mason, William Maynard, Neil McCartan, Caroline M. Moore, Charlotte L. Moss, Kinnari Naik, Anwar Padhani, Chris Parker, Peter Parker, Shonit Punwani, Nahian Rahman, Francesca Rawlins, Manue Rodriguez-Justo, Boris Ruwe, Aida Santaolalla, Harbir Sidhu, Pirruntha Sivaharan, Kamilla Sychowska, Henry Tam, Dizem Tekin, Suparna Thakali, Steve Tuck, Mieke Van Hemelrijck, Hayley Whitaker, Norman Williams, Anna Wingate.

## Data Availability

The datasets generated for this study can be found in the ReIMAGINE instance of the Philips HSDP CDL at https://research-cdl-prod-cdlux.eu-west.philips-healthsuite.com/catalog. These datasets are currently accessible only to the consortium partners of the ReIMAGINE project. After completion of the project, the data will also be available to the broader clinical and scientific community via request to the ReIMAGINE Biological Research Committee (BRC).
